# Magnesium treatment increases gut microbiome synthesizing vitamin D and inhibiting colorectal cancer: results from a double-blind precision-based randomized placebo-controlled trial

**DOI:** 10.1016/j.ajcnut.2025.09.011

**Published:** 2025-09-09

**Authors:** Elizabeth Sun, Xiangzhu Zhu, Reid M Ness, Harvey J Murff, Shan Sun, Chang Yu, Lei Fan, M. Andrea Azcarate-Peril, Martha J Shrubsole, Qi Dai

**Affiliations:** 1 Vanderbilt University School of Medicine, Nashville, TN, United States; 2 Department of Medicine, Division of Epidemiology, Vanderbilt Epidemiology Center, Vanderbilt University School of Medicine, Vanderbilt-Ingram Cancer Center, Vanderbilt University Medical Center, Nashville, TN, United States; 3 Division of Gastroenterology, Hepatology, and Nutrition, Department of Medicine, Vanderbilt University Medical Center, Nashville, TN, United States; 4 Division of Geriatric Medicine, Department of Medicine, Vanderbilt University Medical Center, Nashville, TN, United States; 5 Department of Bioinformatics and Genomics, University of North Carolina at Charlotte, Charlotte, NC, United States; 6 Division of Biostatistics, Department of Population Health, NYU Grossman School of Medicine, New York, NY, United States; 7 Division of Gastroenterology and Hepatology, Department of Medicine, UNC School of Medicine, Chapel Hill, NC, United States

**Keywords:** microbiome, randomized controlled trial, magnesium treatment, colorectal cancer, precision medicine

## Abstract

**Background:**

*Carnobacterium maltaromaticum* and *Faecalibacterium prausnitzii* induce de novo gut synthesis of vitamin D to inhibit colorectal carcinogenesis in mice. Magnesium (Mg) treatment increases circulating vitamin D, and Mg homeostasis is dependent on *TRPM7* genotype.

**Objectives:**

We hypothesize that Mg treatment increases gut *C. maltaromaticum* and *F. prausnitzii,* and the effect differs by *TRPM7* polymorphism.

**Methods:**

The Personalized Prevention of Colorectal Cancer Trial is a double-blind, precision-based randomized controlled trial with 240 participants randomly assigned to both treatment and *TRPM7* genotype. Stool, rectal swabs, and rectal mucosa were collected.

**Results:**

Of 239 participants who completed the trial, 226 with valid microbiome data were analyzed (treatment *n* = 112, placebo *n* = 114). The interaction between treatment and *TRPM7* genotype was only significant for *C. maltaromaticum* (*P* = 0.001) and *F. prausnitzii* (*P* = 0.02) in rectal swabs. In a stratified analysis by *TRPM7* genotype without the missense variant, Mg treatment compared with placebo significantly increased abundance of *C. maltaromaticum* (0.217 ± 0.615 (23.01%) compared with –0.065 ± 0.588 (–6.30%); *P* = 0.006) and *F. prausnitzii* (0.105 ± 0.817 (2.13%) compared with –0.095 ± 0.856 (–1.92%); *P* = 0.04) in rectal swabs. The effect on *C. maltaromaticum* remained after multiple comparisons (*Q* = 0.05 for *C. maltaromaticum* across all sample types and genotypes). In those with the *TRPM7* missense variant, Mg decreased *C. maltaromaticum*, but not *F. prausnitzii,* compared with placebo in rectal swabs [–0.065 ± 0.511 (–6.54%) compared with 0.133 ± 0.503 (13.30%); adjusted *P* = 0.04]**.** The effect did not remain after false discovery rate correction. Mg treatment’s effect on *C. maltaromaticum* in rectal swabs primarily appeared in females, and the treatment-genotype interaction remained significant.

**Conclusions:**

In individuals with adequate *TRPM7* function, Mg supplementation increases the abundance of *C. maltaromaticum* and *F. prausnitzii*.

**Clinical Trial Registry:**

This trial was registered at clinicaltrials.gov as NCT04229992 (https://clinicaltrials.gov/study/NCT04229992?term=NCT04229992&rank=1). The parent study is registered as NCT03265483, and another relevant study is registered as NCT01105169.

## Introduction

Despite a reduction in the incidence of colorectal cancer (CRC) due to increased endoscopic surveillance, CRC remains the fourth most common incident cancer in the United States [1,2]. Growing evidence suggests that the gut microbiome plays a critical role in colorectal carcinogenesis [3,4]. Recently, Li et al. [5] reported that *Carnobacterium maltaromaticum*, a lactic acid bacterium found in the gastrointestinal tract, is depleted in females with CRC [6]. Although it is known that UV radiation via sunlight is essential to vitamin D synthesis in humans [7], Li et al. [5] found that in mice, vitamin D can be synthesized in the absence of sunlight by *C. maltaromaticum* and other microbes, notably *Faecalibacterium prausnitzii,* which worked synergistically to induce de novo gut synthesis of vitamin D metabolites, activate colonic mucosal vitamin D receptor signaling, and inhibit CRC development. Metabolic cross-feeding *of C. maltaromaticum with F*. *prausnitzii converted vitamin D metabolites produced by C. maltaromaticum to active vitamin D* [5]*. F. prausnitzii* has also been associated with inflammatory bowel disease [8,9], irritable bowel syndrome [10], metabolic disease [11], and depression prevention and treatment [12,13].

We previously reported that magnesium (Mg) supplementation significantly increases circulating levels of vitamin D when baseline levels are low [14,15]. This finding was confirmed in subsequent trials [[16], [17], [18], [19]]. Although the mechanism by which this occurs is not fully understood, 1 explanation includes Mg being a cofactor for all the enzymes involved in vitamin D synthesis and metabolism [14,19]. Previous studies suggest that gut concentrations of Mg also play a critical role in regulating the growth and activity of microbiota [[20], [21], [22], [23], [24], [25], [26]]. Thus, in addition to enhancing bodily vitamin D synthesis and metabolism enzymes [15,19], we hypothesize that Mg treatment increases the abundance of *C. maltaromaticum* and *F. prausnitzii* in the gut compared with levels in the placebo arm, which may, in turn, increase de novo vitamin D synthesis and inhibit colorectal carcinogenesis [5]. Other proposed mechanisms by which Mg may modulate CRC risk have included improved insulin sensitivity [27] and promotion of colorectal adenocarcinoma cell apoptosis [28].

*Transient receptor potential cation channel, subfamily M, member 7* (*TRPM7*) encodes a ubiquitously expressed cation channel essential to Mg homeostasis [29]. The inverse association between higher Mg intake and risk of colorectal adenoma differs by *TRPM7* polymorphism [30]. Therefore, we hypothesize that the effect of personalized Mg treatment on gut microbes differs by *TRPM7* genetic polymorphism. Additionally, we hypothesize that *C. maltaromaticum* mediates the effect of Mg treatment on circulating levels of vitamin D. We test these hypotheses within the Personalized Prevention of Colorectal Cancer Trial (PPCCT)*,* a double-blind, precision-based randomized controlled trial (RCT) which was designed to test the interaction between Mg treatment and *TRPM7* genotype on gut carcinogenic biomarkers [14,15]. We also conducted an exploratory analysis to examine whether *C. maltaromaticum and F. prausnitzii are associated with risk of developing metachronous polyps.*

## Methods

### Study population

The PPCCT is a double-blind 2 × 2 factorial precision-based RCT conducted from 2011 to 2016 at Vanderbilt University Medical Center (VUMC) in Nashville, Tennessee. The trial was approved by the VUMC Institutional Review Board (IRB #100106) and was registered on clinicaltrials.gov (NCT04229992). All participants provided written informed consent before participating in the study. Demographic information was collected. Race was self-reported by participants who were asked to select from the following on the initial questionnaire that best described them: White, Black, Asian, Alaskan/Indian, Pacific Islander, Other, Don’t Know, Decline to Answer. The detailed methods were previously reported [15]. In brief, eligible subjects were enrolled sequentially and randomly assigned in blocks of 2 or 4 with a 1:1 ratio to 2 treatment arms, Mg treatment or placebo, within 3 strata defined by the *TRPM7* genotype (GG/GA/AA), a polymorphism at rs8042919 which results in a missense variant [Thr-1482 to isoleucine (Ile)]. Of note, no AA participants were identified despite significant efforts to recruit participants with the AA *TRPM7* genotype. Therefore, only GG and GA genotypes were present in the analysis.

Precision nutrition is an approach to optimize nutrition based on making targeted nutritional recommendations based on an individual’s unique characteristics (i.e., genotype and background nutrition) [31]. In this precision-based trial, each participant in the treatment arm was assigned an individualized daily dose of oral magnesium glycinate for 12 wk that reduced their calcium-to-magnesium ratio to ∼2.3 based on baseline calcium and Mg intake obtained from 2 24-h dietary recalls [15]. Dietary recall assessments were interviewer-administered via telephone for each participant, with 1 on a weekday and 1 on a weekend day if possible. Participants also completed 2 dietary recalls at weeks 1–6 and 2 at weeks 7–12 of the study period. During the dietary assessment, information on participants’ use of medications, nutritional supplementation (vitamins, minerals, fiber, etc.), and other medical conditions was also collected. The Minnesota Nutrient Data System for Research was utilized to collect dietary data and calculate nutrient scores.

**FIGURE 1:**
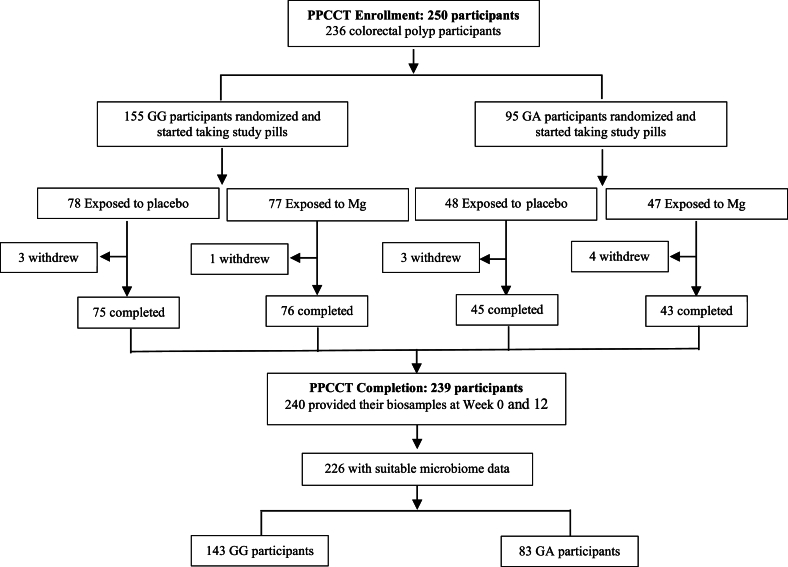
Study population flow diagram (CONSORT). The study population in the Personalized Prevention of Colorectal Cancer Trial: 250 participants were randomly assigned and stared the intervention. Finally, 239 participants completed the study, 240 provided study samples at week 0 and week 12, and 226 had microbiome data suitable for analysis. Mg, magnesium; PPCCT, Personalized Prevention of Colorectal Cancer Trial.

Participants, study investigators, and staff were blinded to the assigned interventions. Blinding was implemented through the Vanderbilt Investigational Drug Service (VIDS). A research pharmacist at VIDS maintained the randomization schedule and was the only person who was aware of the actual interventions. The primary aim of the PPCCT (parent trial) was to examine the effects of Mg supplementation and the interaction with the Mg-*TRPM7* genotype on the expression of biomarkers in the colorectum. The current study, based on a funded R01 project, was separately planned in parallel to the parent study with its own primary aims. At the time of planning, the sample size (*n* = 240 for those who completed the parent trial and donated biospecimens before and after the treatment) was already determined based on the design of the parent study. Thus, the focus of the current study was to estimate the effect size as stated in the primary aims for the funded R01 project on the gut microbiome. It is implicitly understood that, if the effect size is larger than that used for sample size planning in the parent study, the current study will have greater statistical power than the parent study; otherwise, the power would be lower. Two hundred forty participants provided samples and 239 participants completed the study in its entirety. After quality control of the microbiome data at baseline and at the end of the trial, 14 participants were excluded, leaving 226 with valid microbiome data for analysis in the current study (Figure 1).

### Sample collection

Three different biospecimens were collected to measure the gut microbiome. The decision was made to collect stool, rectal mucosa, and rectal swab samples to best represent luminal and adherent microbiota populations, as it has previously been reported that microbial composition varies across sample types, reflecting the varying microhabitats within the digestive tract [32,33]. Stool was collected ≤3 d prior to a study visit by study participants at home using a white plastic collection container covering the toilet bowl, aliquoted by the participant into sterile cryovials, and stored in a home freezer until transport with an ice pack to the study visit. Rectal swabs and mucosal tissues were collected by the study physician at the study visits. Rectal swabs were collected by inserting a culturette swab through the anal canal, swabbing the distal rectal mucosa, and placing the swab into a cryovial. Rectal mucosal samples were collected through an anoscope using standard mucosal biopsy forceps, and these samples were placed into separate storage vials. All 3 biospecimen types were frozen at − 80 °C until use.

Blood samples were collected at each study visit, with participants having fasted ≥8 h. Serum and plasma were rapidly cooled and frozen at –80°C. 25-Hydroxyvitamin D_2_ [25(OH)D_2_] and 25-hydroxyvitamin D_3_ [25(OH)D_3_] were extracted from plasma via liquid–liquid extraction. Then, liquid chromatography–mass spectrometry analysis was conducted.

DNA was extracted from buffy coat fractions or cheek cells from mouthwash samples using a QIAamp DNA mini-kit (Qiagen Inc.). *TRPM7* polymorphism was evaluated with the TaqMan genotyping assay (Assay ID: C_25756319_10; Applied Biosystems).

### Covariates

Information on sociodemographic factors and lifestyle factors at baseline was obtained via questionnaire-based interviews and examination. Sociodemographic factors included age, sex, race/ethnicity, and education level. Race was categorized as White or Black. Education level was separated into 2 levels: less than high school or high school or equivalent, and college or above. Lifestyle factors included consumption of cigarettes and alcohol, physical activity level, and daily intakes of nutrients and foods. Smoking status was classified as never smoker, former smoker, or current smoker. Current alcohol use was defined as ≥1 drink a week over the past 12 mo. Former drinkers did not meet the criterion for current use in the past 12 mo or longer. Physically active was defined as nonoccupational exercise for ≥30 min/d for ≥2 d in the past 7 d. The mean recorded intake from the 2 24-h dietary recalls was used to estimate the baseline daily intake of total energy (kilocalories). Weight was taken on a digital scale and measured in kilograms, and standing height was measured in centimeters with a fixed stadiometer with a vertical backboard and a movable headboard at least twice at each clinic visit. BMI was calculated as weight (kilograms)/height (meters squared).

### Whole genome shotgun (WGS) sequencing of stool, swab, and rectal biopsies

Samples were transferred to a 2 mL tube containing 200 mg ≤106 μm glass beads (Sigma) and 0.3 mL of Qiagen ATL buffer (Qiagen), supplemented with lysozyme (20 mg/mL) (Thermo Fisher Scientific). The suspension was incubated at 37°C for 1 h with occasional agitation and then supplemented with 600 IU of proteinase K and incubated at 60°C for 1 h. Finally, 0.3 mL of Qiagen AL (Qiagen) buffer was added and incubated at 70°C for 10 min, followed with 3 min bead beating in a Qiagen TissueLyser II (Qiagen) at 30 Hz. After a brief centrifugation, supernatants were transferred to a new tube containing 0.3 mL of ethanol. DNA was purified using a standard on-column purification method with Qiagen buffers AW1 and AW2 (Qiagen) as washing agents and eluted in 10 mM Tris (pH 8.0).

WGS sequencing was performed as previously described [32,33]. 10 ng of genomic DNA was processed using the Illumina Nextera XT DNA Sample Preparation Kit (Illumina). Fragmented and tagged DNA was amplified using a limited-cycle PCR program. In this step, index 1 (i7) and index 2 (i5) were added between the downstream bPCR adaptor and the core sequencing library adaptor, as well as primer sequences required for cluster formation. The DNA library was purified using Agencourt AMPure XP Reagent (Beckman Coulter). The DNA library pool was loaded on the Illumina platform reagent cartridge and on the Illumina HiSeq instrument (Illumina). ZymoBIOMICS Microbial Community DNA Standard (Zymo Research Corporation, Cat#D6305) and a library blank composed of library preparation reagents alone were used as controls. Unlike conventional assays that may depend on specific quantification thresholds, whole genome sequencing generally produces extensive data capable of analysis without strict detection limits.

To validate the DNA extraction process, a known bacterial community, the ZymoBIOMICS Microbial Community Standard (Cat# D6300), and reagent-only blanks were processed alongside samples during DNA extraction and library preparation.

Additionally, library blanks containing only library preparation reagents were included to ensure procedural integrity.

### Metachronous colorectal polyp

For the study participants who provided consent for future studies, we reviewed their follow-up colonoscopy results via electronic medical records at VUMC from 2017 to 2018. Information on the colonoscopy or surgery, the diagnosis from the endoscopic mucosal resection procedure, and pathology reports were abstracted. Among 236 participants with a history of colorectal polyps, 124 underwent follow-up colonoscopies after completing the trial with a 3.5-y median follow-up time. The participants were classified as metachronous polyp cases or free of polyps (controls), based on their follow-up colonoscopy and pathology reports. The metachronous cases were further subdivided into metachronous adenomas (tubular, tubulovillous, or villous adenomas) and metachronous serrated polyps (sessile serrated lesions or hyperplastic polyps). The data were used for the exploratory analysis.

### Analysis of shotgun metagenome sequencing

The metagenome sequences were processed with KneadData for quality control and removal of human DNA contamination. MetaPhlAn2 was used for analyzing the taxonomic profiles of shotgun metagenome sequences [34], whereas HUMAnN2 was used for functional profiling including the abundance of functional pathways [35]. Both taxonomy-stratified and unstratified pathway abundance were generated. In the stratified abundance table, the abundance of each pathway was shown as abundance in each taxon, whereas in the unstratified abundance table, the abundance of each pathway was not separated by taxa. The abundance of taxa and pathways was normalized as below to correct for different sequencing depth [32,33].

$$N_{ij\_\text{norm}}=\log10\left(\frac{N_{ij}}{N_{\text{sample}\_j}}*N_{\text{avg}}+1\right)$$

*N*_*ij*_ is the raw abundance of taxa or pathway *i* in sample *j*. *N*_sample_*j*_ is the total number of sequences in sample *j*. *N*_avg_ is the average number of reads per sample. *N*_*ij*_norm_ is the normalized abundance of taxa or pathway *i* in sample *j*.

### Statistical analyses

Baseline characteristics for continuous variables (mean ± SD) and categorical variables (count and percent) were reported (Table 1). To assess the effect of personalized Mg treatment on changes in the abundance of *F. prausnitzii* or *C. maltaromaticum*, generalized linear models were used. On the basis of the PPCCT’s original study design, the main effect and the effect stratified by the *TRPM7* genotype were considered as primary analyses. Other stratified analyses were secondary. Unadjusted models and models adjusting for age, sex, race, and baseline levels were evaluated. Stratified analyses by *TRPM7* genotype (GG, GA) and/or sex (male or female) were conducted. Mediation analyses were conducted to examine whether the effect of Mg treatment on circulating vitamin D metabolites was mediated by *C. maltaromaticum* and *F. prausnitzii.* In the exploratory analysis, to study the associations between the abundance of microbiota and risk of metachronous colorectal adenoma/serrated polyp, logistic regression models were employed to estimate the odds ratios (ORs) and 95% confidence intervals (CIs) without adjusting (model 1) or adjusting for age, sex, and BMI (model 2). The abundances of *F. prausnitzii* or *C. maltaromaticum* were categorized into tertiles based on the distribution of the controls in all analyses. Tests for trends were performed in the models using the lowest tertile level as the reference group and considering the tertile as a continuous variable in the model.

**TABLE 1:** Descriptive characteristics of the study population at baseline.

	Magnesium (*N* = 120)	Placebo (*N* = 120)
Age	60.2 ± 7.8	61.2 ± 8.1
Sex: males (%)	65 (54)	62 (52)
Race: White (%)	118 (98.3)	119 (99.2)
Race: African American (%)	2 (1.7)	1 (0.8)
BMI	29.9 ± 6.1	30.6 ± 6.6
Education: college and above	108 (90.0)	108 (90.0)
*TRPM*7 genotype (%)		
GG	77 (64)	75 (62)
GA	43 (36)	45 (38)
Smoking status (%)		
Never	59 (49)	72 (60)
Former	51 (42)	40 (33)
Current	10 (8)	8 (7)
Drinking status (%)		
Never	39 (32)	51 (42)
Former	25 (21)	24 (20)
Current	56 (47)	45 (38)
Physically active ≥2 d/wk (%)	93 (77)	102 (85)
Less than a college education (%)	12 (10)	12 (10)
Total energy intake (kcal/d)	2084 ± 547	2108 ± 604
Total magnesium intake (mg/d)	364 ± 97	338 ± 99
Total vitamin D intake (μg/d)	22 ± 21	42 ± 162
Ca:Mg ratio at baseline	3.7 ± 0.9	3.9 ± 1.5
Ca:Mg ratio during 12-wk trial	2.1 ± 0.7	3.5 ± 1.3
Plasma vitamin D level		
25(OH)D (ng/mL)	33.4 ± 10.2	33.0 ± 12.7
25(OH)D_3_ (ng/mL)	32.3 ± 10.4	30.1 ± 11.3
25(OH)D_2_ (ng/mL)	1.1 ± 2.3	2.8 ± 10.9
24,25(OH)_2_D_3_ (ng/mL)	4.7 ± 3.7	3.8 ± 2.8
Baseline relative abundance		
C. malt*aromaticum*		
Rectal mucosa	0.101 ± 0.200	0.139 ± 0.220
Rectal swab	0.959 ± 0.537	1.021 ± 0.443
Stool	1.185 ± 0.448	1.204 ± 0.411
*F. prausnitzii*		
Rectal mucosa	3.441 ± 0.400	3.448 ± 0.330
Rectal swab	4.954 ± 0.783	4.991 ± 0.702
Stool	5.384 ± 0.529	5.328 ± 0.573

Continuous variables are presented as mean ± SD.

Abbreviations: 25(OH)D, 25-hydroxyvitamin D; 25(OH)D_3_, 25-hydroxyvitamin D_3_; 25(OH)D_2_; 25-Hydroxyvitamin D_2_; 24,25(OH)_2_D_3_, 24,25-dihydroxyvitamin D3; Ca, calcium; *C. maltaromaticum*, *Carnobacterium maltaromaticum; F. prausnitzii, Faecalibacterium prausnitzii*; Mg, magnesium; *TRPM7, transient receptor potential cation channel, subfamily M, member 7*.

All *P* values are 2 sided and statistical significance was determined using an *α* level of 0.05. To account for multiple comparisons, false discovery rate (FDR) correction was applied to primary analyses in TABLE 2, TABLE 3, respectively, comparing the efficacy of Mg treatment on every bacterium across different sample types and different genotypes. Statistical significance for FDR correction was determined using a Q level of 0.1, given the exploratory nature of the investigation. The data analyses were performed with software SAS Enterprise Guide 7.1 (version 9.4; SAS Institute).

**TABLE 2:** Changes in *C. maltaromaticum relative abundance* by magnesium compared with placebo in rectal mucosa, swab, and stool.

Participant category and sample type	Change in *C. maltaromaticum* relative abundance from baseline						FDR correction (*Q*)
	Mg treatment		Placebo		*P*_1_	*P*_2_	
	Relative abundance (mean ± SD)	%	Relative abundance (mean ± SD)	%			
All participants (*n* = 226)							
Rectal mucosa	0.001 ± 0.268	0.64	–0.010 ± 0.294	–7.04	0.78	0.50	0.56
Rectal swab	0.110 ± 0.592	11.49	0.013 ± 0.562	1.24	0.23	0.31	0.56
Stool	0.051 ± 0.397	4.32	0.009 ± 0.459	0.73	0.46	0.46	0.56
*TRPM7* genotype: GG (*n* = 143)							
Rectal mucosa	0.007 ± 0.249	7.92	–0.027 ± 0.286	–19.30	0.44	0.93	0.93
Rectal swab	0.217 ± 0.615	23.01	–0.065 ± 0.588	–6.30	0.009	0.006	0.05
Stool	0.102 ± 0.416	8.83	–0.018 ± 0.446	–1.48	0.11	0.12	0.36
*TRPM7* genotype: GA (*n* = 83)							
Rectal mucosa	–0.011 ± 0.301	–9.41	0.021 ± 0.308	15.53	0.64	0.36	0.56
Rectal swab	–0.065 ± 0.511	–6.54	0.133 ± 0.503	13.30	0.09	0.04	0.18
Stool	–0.042 ± 0.346	–3.33	0.050 ± 0.481	4.02	0.33	0.48	0.56

Generalized linear models were used: *P*1 not adjusted*; P*2 adjusted for age, sex, BMI, and baseline level.

*P* for interaction between treatment and genotype of *TRPM7 for C. maltaromaticum*: 0.55 in mucosa; 0.001 in swab; and 0.11 in stool.

Abbreviations: *C. maltaromaticum*, *Carnobacterium maltaromaticum*; FDR, false discovery rate; Mg, magnesium; *TRPM7, transient receptor potential cation channel, subfamily M, member 7.*

**TABLE 3:** Changes in *F. prausnitzii* relative abundance by magnesium compared with placebo in rectal mucosa, swab, and stool.

Participant category and sample type	Change in *F. prausnitzii* relative abundance from baseline						FDR correction (*Q*)
	Mg treatment		Placebo		*P*_1_	*P*_2_	
	(mean ± SD)	%	(mean ± SD)	%			
All participants (*n* = 226)							
Rectal mucosa	–0.017 ± 0.392	–0.49	0.046 ± 0.425	1.34	0.25	0.16	0.36
Rectal swab	0.044 ± 0.787	0.89	–0.028 ± 0.752	–0.56	0.48	0.48	0.86
Stool	–0.073 ± 0.469	–1.35	–0.047 ± 0.558	–1.88	0.71	0.91	0.91
*TRPM7* genotype: GG (*n* = 143)							
Rectal mucosa	0.006 ± 0.315	0.18	–0.012 ± 0.369	–0.34	0.75	0.79	0.89
Rectal swab	0.105 ± 0.817	2.13	–0.095 ± 0.856	–1.92	0.15	0.04	0.18
Stool	–0.082 ± 0.518	–1.52	–0.051 ± 0.565	–0.98	0.73	0.77	0.89
*TRPM7* genotype: GA (*n* = 83)							
Rectal mucosa	–0.058 ± 0.503	–1.66	0.145 ± 0.495	4.17	0.07	0.01	0.09
Rectal swab	–0.060 ± 0.730	–1.20	0.087 ± 0.516	1.72	0.28	0.12	0.36
Stool	–0.057 ± 0.375	–1.06	–0.040 ± 0.554	–0.74	0.87	0.67	0.89

Generalized linear models were used: *P*1 not adjusted*; P*2 adjusted for age, sex, BMI, and baseline level.

*P* for interaction between treatment and genotype of *TRPM7 for F. prausnitzii:* 0.01 in mucosa; 0.02 in swab; and 0.61 in stool.

Abbreviations: FDR, false discovery rate; *F. prausnitzii, Faecalibacterium prausnitzii*; Mg, magnesium; *TRPM7, transient receptor potential cation channel, subfamily M, member 7*.

## Results

The current analysis includes 226 participants. The participants in the Mg treatment arm did not differ significantly compared with those in the placebo arm in terms of age, sex, race, BMI, *TRPM7* genotype (GG or GA), smoking status, drinking status, physical activity, education level, and daily total energy intake (Table 1). The average ages were 60.2 and 61.2 y in the treatment and placebo arms, respectively. Of 108 female participants, 40.7% (*n* = 44) carried the GA genotype. Of 118 male participants, 33.0% (*n* = 39) carried the GA genotype.

Baseline relative abundances of *C. maltaromaticum* and *F. prausnitzii* for treatment and placebo groups are shown in Table 1. In rectal swab samples at baseline (Supplemental Table 1), females had a significantly higher relative abundance of *C. maltaromaticum* (1.103 ± 0.489, mean ± SD) compared with males (0.885 ± 0.471) (*P* = 0.001 for difference). There were no significant differences in baseline relative abundances of *C. maltaromaticum* or *F. prausnitzii* between males and females in rectal mucosa and stool samples. As primary analyses, pre- to posttreatment changes in *C. maltaromaticum* relative abundance are shown in Table 2. Overall, the changes in *C. maltaromaticum* abundance did not significantly differ between the Mg treatment and the placebo groups for any sample type. However, when stratified by *TRPM7* genotype*,* those with the GG genotype (i.e., without the missense variant) in the treatment arm had a significant increase in *C. maltaromaticum* abundance compared with placebo in rectal swab samples [0.217 ± 0.615 (23.01%) compared with –0.065 ± 0.588 (–6.30%); adjusted *P* = 0.006]*,* whereas those with the *TRPM7* missense variant (GA) had a significant decrease in *C. maltaromaticum* abundance in the treatment arm compared with placebo in rectal swab samples [–0.065 ± 0.511 (–6.54%) compared with 0.133 ± 0.503 (13.30%); adjusted *P* = 0.04]. After FDR correction, the increased effect of personalized Mg treatment in those with the *TRPM7* GG genotype remained significant (*Q* = 0.05), whereas the observed decrease in those with the *TRPM7* GA genotype did not remain significant (*Q* = 0.18). The test for the interaction between the treatment and the *TRPM7* genotype was statistically significant (*P* = 0.001) and remained significant in females (*P* = 0.01). As secondary analyses, changes in *C. maltaromaticum* relative abundance stratified by sex alone and sex and *TRPM7* genotype are shown in Supplemental Table 2. When stratified by sex alone, there were no significant changes in *C. maltaromaticum* compared with placebo. However, when stratified by both sex and *TRPM7* genotype, females with the GG genotype who had received Mg treatment had a significantly higher increase in abundance of *C. maltaromaticum* compared with placebo in rectal swab samples [0.239 ± 0.624 (24.06%) compared with –0.177 ± 0.588 (–14.70%); adjusted *P* = 0.02], and stool samples [0.152 ± 0.406 (13.52%) compared with –0.109 ± 0.513 (–9.27%); adjusted *P* = 0.04]. There were no significant changes in *C. maltaromaticum* among females with the *TRPM7* GA or males with the GG and the GA genotypes when comparing Mg treatment compared with placebo groups. No significant effect was identified in the analysis of rectal mucosa.

As primary analyses, the changes in abundance of *F. prausnitzii* did not change significantly in the Mg treatment group compared with the placebo group (Table 3). In a stratified analysis by *TRPM7* genotype, participants with the GG genotype in the Mg treatment group had a significant increase in *F. prausnitzii* abundance in rectal swab samples compared with the placebo group [0.105 ± 0.817 (2.13%) compared with –0.095 ± 0.856 (–1.92%); adjusted *P* = 0.04], whereas participants with the GA genotype in the Mg treatment group tended to have a decrease in *F. prausnitzii* abundance in rectal swab samples compared with the placebo group [–0.060 ± 0.730 (–1.20%) compared with –0.087 ± 0.516 (1.72%); borderline significance with adjusted *P* = 0.12]. Notably, the observed increase in *F. prausnitzii* abundance in rectal swab samples did not remain significant after FDR correction was applied (*Q* = 0.18). The test for the interaction between the treatment and the *TRPM7* genotype was statistically significant (*P* = 0.02) in the analysis of rectal swabs. The participants with the GA genotype in the Mg treatment group had a significant decrease in *F. prausnitzii* abundance in rectal mucosa compared with the placebo group [–0.058 ± 0.503 (–1.66%) compared with 0.145 ± 0.495 (4.17%); adjusted *P* = 0.01, *Q* = 0.09] whereas those with the GG genotype in the Mg treatment arm did not have an apparent change in *F. prausnitzii* abundance in rectal mucosa compared with the placebo group [0.006 ± 0.315 (0.18%) compared with –0.012 ± 0.369 (–0.34%); adjusted *P* = 0.79]. The test for the interaction between the treatment and the *TRPM7* genotype was statistically significant (*P* = 0.01) in the analysis of rectal mucosa. As secondary analyses, no significant changes in *F. prausnitzii* abundance were observed in the Mg treatment compared with the placebo groups when stratified by sex alone, or *TRPM7* genotype combined with sex (Supplemental Table 3). No significant effect was identified in the analysis of stool samples.

A mediation analysis was performed to investigate whether the changes in abundance of *C. maltaromaticum* or *F. prausnitzii* mediate the effect of Mg treatment on alterations in circulating levels of vitamin D. No significant mediation effect was identified. Additional stratified analyses by baseline and changes of 25(OH)D levels showed no significant microbe-mediated effects of Mg treatment on vitamin D metabolites. We also did not find any relationship between plasma 25(OH)D metabolites and changes in abundance in *C. maltaromaticum* and *F. prausnitzii* (data not shown).

An exploratory data analysis was also performed to examine the associations between levels of *C. maltaromaticum* and *F. prausnitzii* and risk of polyp development. In rectal mucosa, a higher abundance of *F. prausnitzii* was significantly associated with an almost 3-fold increased risk of developing metachronous polyps [OR = 2.84 (1.05, 7.65), adjusted *P* = 0.04]. Meanwhile, in rectal swabs, higher levels of *C. maltaromaticum* were marginally associated with a decreased risk of developing serrated polyps [OR = 0.15 (0.02, 1.38), adjusted *P =* 0.10]. In all other sample types, *C. maltaromaticum* and *F. prausnitzii* abundance were not associated with risk of polyp development (Table 4).

**TABLE 4:** Odds ratios (ORs) and 95% CIs for risk of metachronous colorectal polyps by relative abundance of bacteria in mucosal tissue and swab, results from the PPCCT.

Bacteria	Cases/controls	Model	Tertile relative abundance			*P* trend
			Ref	OR (95% CI)	OR (95% CI) (high)	
Rectal mucosa						
Metachronous polyp						
C. malt*aromaticum*	68/53	Model 1	1.00	0.71 (0.32, 1.60)		0.41
C. malt*aromaticum*	68/53	Model 2	1.00	0.83 (0.35, 1.96)		0.66
F. p*rausnitzii*	68/53	Model 1	1.00	2.00 (0.77, 5.18)	2.82 (1.11, 7.21)	0.03
F. p*rausnitzii*	68/53	Model 2	1.00	1.71 (0.62, 4.71)	2.84 (1.05, 7.65)	0.04
Metachronous adenoma						
C. malt*aromaticum*	41/53	Model 1	1.00	0.56 (0.21, 1.48)		0.24
C. malt*aromaticum*	41/53	Model 2	1.00	0.61 (0.21, 1.73)		0.35
F. p*rausnitzii*	41/53	Model 1	1.00	2.11 (0.76, 5.90)	1.53 (0.52, 4.49)	0.47
F. p*rausnitzii*	41/53	Model 2	1.00	1.88 (0.62, 5.64)	1.38 (0.42, 4.46)	0.62
Serrated polyp						
C. malt*aromaticum*	14/53	Model 1	1.00	0.93 (0.25, 3.39)		0.91
C. malt*aromaticum*	14/53	Model 2	1.00	0.97 (0.26, 3.67)		0.96
F. p*rausnitzii*	14/53	Model 1	1.00	2.50 (0.43, 14.60)	3.71 (0.67, 20.40)	0.13
F. p*rausnitzii*	14/53	Model 2	1.00	2.11 (0.34, 12.96)	3.41 (0.60, 19.50)	0.16
Rectal swab						
Metachronous polyp						
C. malt*aromaticum*	69/53	Model 1	1.00	0.77 (0.33, 1.79)	0.56 (0.23, 1.39)	0.21
C. malt*aromaticum*	69/53	Model 2	1.00	0.75 (0.30, 1.85)	0.68 (0.26, 1.79)	0.42
F. p*rausnitzii*	69/53	Model 1	1.00	0.71 (0.29, 1.74)	1.24 (0.52, 2.91)	0.62
F. p*rausnitzii*	69/53	Model 2	1.00	0.70 (0.26, 1.86)	1.21 (0.48, 3.02)	0.67
Metachronous adenoma						
C. malt*aromaticum*	42/53	Model 1	1.00	0.63 (0.24, 1.67)	0.61 (0.23, 1.66)	0.31
C. malt*aromaticum*	42/53	Model 2	1.00	0.65 (0.23, 1.87)	0.78 (0.26, 2.35)	0.61
F. p*rausnitzii*	42/53	Model 1	1.00	0.56 (0.20, 1.60)	1.12 (0.43, 2.91)	0.81
F. p*rausnitzii*	42/53	Model 2	1.00	0.63 (0.20, 2.00)	1.31 (0.46, 3.75)	0.61
Serrated polyp						
C. malt*aromaticum*	14/53	Model 1	1.00	0.86 (0.24, 3.06)	0.15 (0.02, 1.36)	0.09
C. malt*aromaticum*	14/53	Model 2	1.00	0.83 (0.20, 3.38)	0.15 (0.02, 1.38)	0.10
F. p*rausnitzii*	14/53	Model 1	1.00	0.80 (0.18, 3.47)	1.06 (0.26, 4.32)	0.94
F. p*rausnitzii*	14/53	Model 2	1.00	0.84 (0.18, 3.94)	0.95 (0.22, 4.03)	0.94
Stool						
Metachronous polyp						
C. malt*aromaticum*	68/50	Model 1	1.00	1.02 (0.41, 2.54)	1.75 (0.73, 4.22)	0.21
C. malt*aromaticum*	68/50	Model 2	1.00	0.81 (0.30, 2.17)	1.56 (0.62, 3.97)	0.35
F. p*rausnitzii*	68/50	Model 1	1.00	0.91 (0.37, 2.25)	1.26 (0.52, 3.05)	0.61
F. p*rausnitzii*	68/50	Model 2	1.00	0.78 (0.29, 2.08)	1.27 (0.49, 3.30)	0.60
Metachronous adenoma						
C. malt*aromaticum*	41/50	Model 1	1.00	0.85 (0.30, 2.42)	1.54 (0.58, 4.09)	0.40
C. malt*aromaticum*	41/50	Model 2	1.00	0.66 (0.21, 2.09)	1.42 (0.49, 4.12)	0.54
F. p*rausnitzii*	41/50	Model 1	1.00	0.93 (0.34, 2.55)	1.06 (0.39, 2.91)	0.91
F. p*rausnitzii*	41/50	Model 2	1.00	0.75 (0.25, 2.29)	1.23 (0.41, 3.72)	0.73
Serrated polyp						
C. malt*aromaticum*	14/50	Model 1	1.00	0.34 (0.06, 1.87)	0.90 (0.24, 3.43)	0.81
C. malt*aromaticum*	14/50	Model 2	1.00	0.28 (0.05, 1.67)	0.74 (0.18, 2.98)	0.63
F. p*rausnitzii*	14/50	Model 1	1.00	0.40 (0.07, 2.35)	1.49 (0.39, 5.65)	0.52
F. p*rausnitzii*	14/50	Model 2	1.00	0.30 (0.05, 2.04)	1.32 (0.30, 5.80)	0.57

Unconditional logistic regression models, model 1, unadjusted; model 2, adjusted for age (continuous), sex, and BMI (continuous).

Metachronous polyps are further classified into metachronous adenomas or metachronous serrated polyps.

Abbreviations: *C. maltaromaticum*, *Carnobacterium maltaromaticum*; CI, confidence interval; *F. prausnitzii, Faecalibacterium prausnitzii*; PPCCT, Personalized Prevention of Colorectal Cancer Trial; ref, reference.

## Discussion

Our findings demonstrate that Mg treatment increases the abundance of *C. maltaromaticum* and likely *F. prausnitzii* in participants without the *TRPM7* missense variant. Li et al. [5] noted increased intestinal vitamin D production in *C. maltaromaticum*-treated mice. In additional experiments, *C. maltaromaticum* led to the increased levels of 7-dehydrocholesterol whereas *F. prausnitzii* converted 7-dehydrocholesterol to 25(OH)D_3_ and 1,25(OH)_2_D_3_ and, in turn, decreased CRC incidence. Our findings in this RCT suggest that Mg treatment increased both *C. maltaromaticum* and *F. prausnitzii*, which can convert vitamin D precursors to 25(OH)D_3_ and 1,25(OH)2D3 in the gut [5].

We previously demonstrated that Mg treatment increases 25(OH)D_3_ when the baseline 25(OH)D level is lower [15]. The mechanism by which this occurs is likely multifactorial. Here, *C. maltaromaticum* and *F. prausnitzii* were not mediators of Mg treatment’s effect on circulating vitamin D levels, suggesting that Mg increases gut microbial abundance and vitamin D levels through independent mechanisms. Thus, Mg supplementation likely increases vitamin D synthesis and metabolism enzymes in the body [15]. Although we cannot eliminate the possibility that statistical power was insufficient to observe an association, we found that Mg treatment significantly increases circulating levels of 25(OH)D_3_ and *C. maltaromaticum* and likely *F*. *prausnitzii* (borderline significance) in rectal swabs compared with placebo. Thus, based on Li et al.’s [5] findings, we postulate that local de novo synthesis of gut vitamin D caused by increased *C. maltaromaticum* and *F. prausnitzii* may only lead to a local effect, including inhibition of colorectal carcinogenesis.

The observed effect of Mg treatment on these microbes is biologically plausible. Mg is essential for many cellular functions in gut microbiota [20,21] and is maintained at high levels (∼0.5–2.0 mM) in microbiota, unlike most divalent cations [21]. Several bacterial metabolic enzymes are Mg-dependent [[22], [23], [24], [25], [26],^36^,^37^], and Mg concentration in culture media is essential for bacterial growth [[38], [39], [40]]. Meanwhile, *TRPM7* is a key ion channel that regulates Mg homeostasis [41,42]. An in vitro study discovered that the heterologously expressed Thr1482Ile missense variant in the *TRPM7* gene caused an elevated sensitivity to inhibition by intracellular Mg^2+^ [43]. We observed that Mg treatment only significantly increased the abundance of *C. maltaromaticum* and *F. prausnitzii* in rectal swabs for those without the missense *TRPM7* genotype. Meanwhile, Mg treatment significantly reduced the abundance of *C. maltaromaticum* in those with the missense variant. Taken together, these findings suggest that Mg treatment in the context of a functional polymorphism in the *TRPM7* gene is key to these microbes’ ability to locally synthesize vitamin D in the gut and may inhibit colorectal carcinogenesis [5].

In our exploratory analysis investigating risk of polyp development, we found that a higher abundance of *C. maltaromaticum* in rectal swabs was marginally associated with a reduced risk of developing serrated polyps, whereas a higher abundance of *F. prausnitzii* in rectal mucosa was significantly associated with an ∼3-fold increased risk of developing metachronous polyps. It has been found that a higher abundance of *F. prausnitzii* in rectal mucosa indicates a loss of gut integrity, which is linked to increased levels of inflammation and progression of colorectal polyps [44]. In our study, Mg treatment reduced *F. prausnitzii* in the rectal mucosa in those with the missense variant. These findings suggest that Mg treatment in missense *TRPM7* individuals may decrease CRC risk and could explain the previous finding that higher intakes of Mg were associated with a reduced risk of colorectal polyps among those with the Thr1482Ile missense variant in the *TRPM7* gene [30].

Li et al. [5] reported that *C. maltaromaticum* was higher in abundance in females compared with males in an in-house cohort and in a public inflammatory bowel disease dataset, and further, *C. maltaromaticum* was depleted in females with CRC [6]. Furthermore, the finding that *C. maltaromaticum* and *F. prausnitzii* could synthesize vitamin D and inhibit colorectal carcinogenesis was shown in female mice. Consistent with this, we found that the abundance of *C. maltaromaticum* was higher in females than in males at baseline. Mg supplementation did not have a significant effect for males, regardless of *TRPM7* genotype, suggesting a sex effect leading to the increased bacterial abundance observed in females. This observed difference could be explained by the known role of estrogen in shifting Mg from circulation into cells, leading to increased cellular uptake in females [45]. Thus, estrogen may play a mediating role in Mg supplementation and its downstream effects.

Interestingly, the only significant increases caused by Mg treatment were observed in rectal swabs in females. This may be due to differences in oxygenation of the sample environment, as the colon is known to have the most drastic oxygen gradient in the body [33]. Anoxia increases markedly from colorectal mucosa to rectal swabs to stool samples [33]. Li et al. [5] reported that *C. maltaromaticum* and *F. prausnitzii* operate synergistically to produce active vitamin D via fermentation. As such, to conduct fermentation in the gut, *C. maltaromaticum* and *F. prausnitzii* may be of higher abundance in low oxygenation environments (i.e., rectal swab and stool samples) and in lower abundance in higher oxygenation environments (i.e., colorectal mucosa).

This study has several strengths. It features a double-blind, precision-based RCT in which participants demonstrated strong adherence to the study protocol, and attrition rates were low. We collected a variety of samples that represented the gastrointestinal microbiome in different niches, giving a better representation of participants’ microbiomes [46]. Notably, our findings align with recent research, including a sex effect associated with increased *C. maltaromaticum* abundance [5]. Furthermore, the PPCCT was specifically designed to test the interaction between Mg treatment and *TRPM7* genotype on gut biomarkers related to colorectal carcinogenesis. Our findings provide support for the *TRPM7* genotype-Mg intake interaction affecting risk of colorectal polyp development [30].

This study also has some limitations. Although the benefits of an increase in *C. maltaromaticum* and *F. prausnitzii* relative abundances are hypothesized to increase de novo synthesize vitamin D and reduce colorectal carcinogenesis, we did not specifically investigate the downstream effects of increased *C. maltaromaticum* and *F. prausnitzii* abundance. Also, although our adjusted *P* values showed significant increases in both *C. maltaromaticum* and *F. prausnitzii*, the significant effect did not remain for *F. prausnitzii* after FDR correction. Specific strains of *C. maltaromaticum and F. prausnitzii* were not distinguished in our study, warranting future investigation into Mg’s effects on vitamin D production among specific strains. Additionally, given that changes in microbe abundance were quantified in relative abundances, changes could have occurred due to other bacteria not studied. Finally, our study population is limited in race, ethnicity, and geographic diversity, limiting the generalizability of our findings.

**FIGURE 2:**
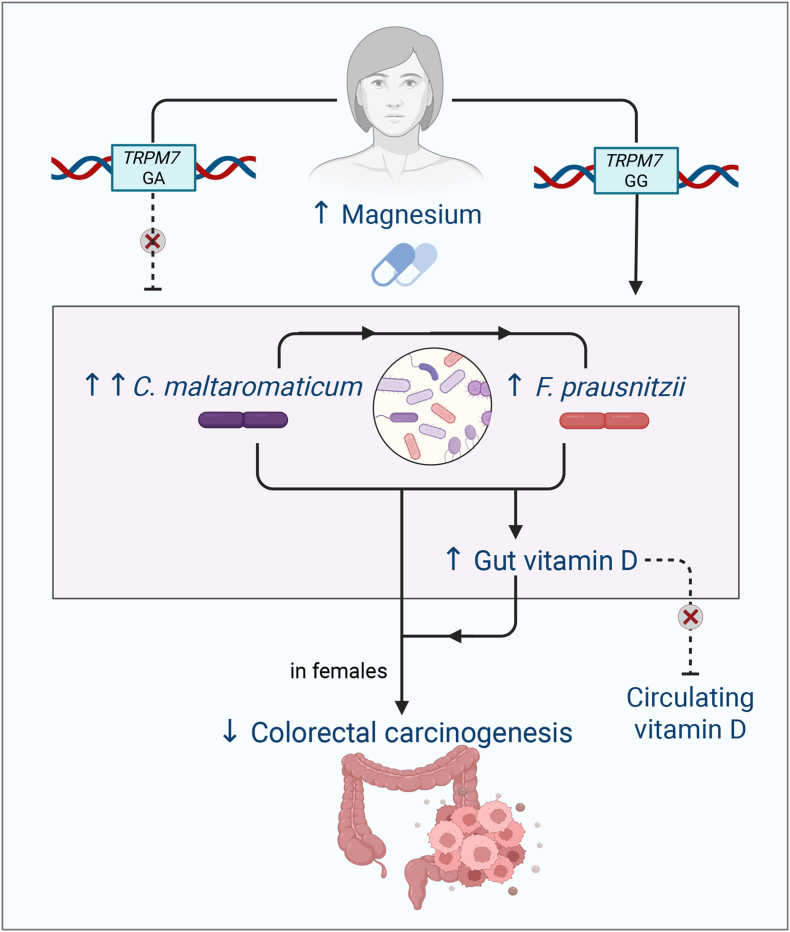
Mechanistic figure: magnesium supplementation increases levels of *C. maltaromaticum* and *F. prausnitzii. TRPM7, transient receptor potential cation channel, subfamily M, member 7*.

In conclusion (Figure 2), we found that Mg supplementation increases the abundance of *C. maltaromaticum* and *F. prausnitzii* in individuals with adequate *TRPM7* function, specifically in females. In those with a missense variant in the *TRPM7* gene, Mg treatment reduces the abundance of *F. prausnitzii* in colorectal mucosa. Li et al. [5] previously revealed that *F. prausnitzii* converted vitamin D precursors produced by *C. maltaromaticum* into active metabolites and inhibited CRC development in females. Together, these findings lay a foundation for a precision-based strategy for the prevention of CRC in high-risk populations.

## Author contributions

The authors’ responsibilities were as follows – MJS, CY, QD: designed research; XZ, MJS, RN, HJM, CY, QD: conducted the research; MAA-P: conducted the assay; ES, XZ, SS: analyzed data; ES, QD: drafted the manuscript; and all authors: contributed to the data interpretation and manuscript revision and approved the final version of this manuscript.

## Data availability

Data described in the manuscript, code book, and analytic code will be made available on request.

## Funding

The authors reported no funding received for this study.

## Conflict of interest

The authors report no conflicts of interest.

## References

[1] Saraiva M.R., Rosa I., Claro I. (2023). Early-onset colorectal cancer: a review of current knowledge. World J. Gastroenterol..

[2] Siegel R.L., Giaquinto A.N., Jemal A. (2024). Cancer statistics, 2024. CA Cancer J. Clin..

[3] Knippel R.J., Drewes J.L., Sears C.L. (2021). The cancer microbiome: recent highlights and knowledge gaps. Cancer Discov.

[4] Iftekhar A., Berger H., Bouznad N., Heuberger J., Boccellato F., Dobrindt U. (2021). Genomic aberrations after short-term exposure to colibactin-producing E. coli transform primary colon epithelial cells. Nat. Commun..

[5] Li Q., Chan H., Liu W.X., Liu C.A., Zhou Y., Huang D. (2023). Carnobacterium maltaromaticum boosts intestinal vitamin D production to suppress colorectal cancer in female mice. Cancer Cell.

[6] Ramia N.E., Mangavel C., Gaiani C., Gueudin A.M., Taha S., Revol-Junelles A.-M. (2020). Nested structure of intraspecific competition network in Carnobacterium maltaromaticum. Sci. Rep..

[7] Webb A.R. (2006). Who, what, where and when—influences on cutaneous vitamin D synthesis. Prog. Biophys. Mol. Biol..

[8] Machiels K., Joossens M., Sabino J., De Preter V., Arijs I., Eeckhaut V. (2014). A decrease of the butyrate-producing species Roseburia hominis and Faecalibacterium prausnitzii defines dysbiosis in patients with ulcerative colitis. Gut.

[9] Sokol H., Pigneur B., Watterlot L., Lakhdari O., Bermúdez-Humarán L.G., Gratadoux J.J. (2008). Faecalibacterium prausnitzii is an anti-inflammatory commensal bacterium identified by gut microbiota analysis of Crohn disease patients. Proc. Natl. Acad. Sci. U. S. A..

[10] Rajilić-Stojanović M., Biagi E., Heilig H.G., Kajander K., Kekkonen R.A., Tims S. (2011). Global and deep molecular analysis of microbiota signatures in fecal samples from patients with irritable bowel syndrome. Gastroenterology.

[11] Karlsson F.H., Tremaroli V., Nookaew I., Bergström G., Behre C.J., Fagerberg B. (2013). Gut metagenome in European women with normal, impaired and diabetic glucose control. Nature.

[12] Hao Z., Wang W., Guo R., Liu H. (2019). Faecalibacterium prausnitzii (ATCC 27766) has preventive and therapeutic effects on chronic unpredictable mild stress-induced depression-like and anxiety-like behavior in rats. Psychoneuroendocrinology.

[13] Samuthpongtorn C., Chan A.A., Ma W., Wang F., Nguyen L.H., Wang D.D. (2024). *F. prausnitzii* potentially modulates the association between citrus intake and depression. Microbiome.

[14] Liu S., Liu Q. (2018). Personalized magnesium intervention to improve vitamin D metabolism: applying a systems approach for precision nutrition in large randomized trials of diverse populations. Am. J. Clin. Nutr..

[15] Dai Q., Zhu X., Manson J.E., Song Y., Li X., Franke A.A. (2018). Magnesium status and supplementation influence vitamin D status and metabolism: results from a randomized trial. Am. J. Clin. Nutr..

[16] Vázquez-Lorente H., Herrera-Quintana L., Molina-López J., Gamarra-Morales Y., López-González B., Miralles-Adell C. (2020). Response of vitamin D after magnesium intervention in a postmenopausal population from the province of Granada, Spain. Nutrients.

[17] Cheung M.M., Dall R.D., Shewokis P.A., Altasan A., Volpe S.L., Amori R. (2022). The effect of combined magnesium and vitamin D supplementation on vitamin D status, systemic inflammation, and blood pressure: a randomized double-blinded controlled trial. Nutrition.

[18] Kisters K., Kisters L., Werner T., Deutsch A., Westhoff T., Gröber U. (2020). Increased serum vitamin D concentration under oral magnesium therapy in elderly hypertensives. Magnes. Res..

[19] Deng X., Song Y., Manson J.E., Signorello L.B., Zhang S.M., Shrubsole M.J. (2013). Magnesium, vitamin D status and mortality: results from US National Health and Nutrition Examination Survey (NHANES) 2001 to 2006 and NHANES III. BMC Med.

[20] Blanc-Potard A.B., Groisman E.A. (2021). How pathogens feel and overcome magnesium limitation when in host tissues. Trends Microbiol.

[21] Shin J.H., Wakeman C.A., Goodson J.R., Rodionov D.A., Freedman B.G., Senger R.S. (2014). Transport of magnesium by a bacterial Nramp-related gene. PLOS Genet.

[22] Beld J., Blatti J.L., Behnke C., Mendez M., Burkart M.D. (2014). Evolution of acyl-ACP-thioesterases and β-ketoacyl-ACP-synthases revealed by protein-protein interactions. J. Appl. Phycol..

[23] Cantu D.C., Chen Y., Reilly P.J. (2010). Thioesterases: a new perspective based on their primary and tertiary structures. Protein Sci.

[24] Libertini L.J., Smith S. (1978). Purification and properties of a thioesterase from lactating rat mammary gland which modifies the product specificity of fatty acid synthetase. J. Biol. Chem..

[25] García-Legorreta A., Soriano-Pérez L.A., Flores-Buendía A.M., Medina-Campos O.N., Noriega L.G., Granados-Portillo O. (2020). Effect of dietary magnesium content on intestinal microbiota of rats. Nutrients.

[26] Lusk J.E., Williams R.J., Kennedy E.P. (1968). Magnesium and the growth of Escherichia coli. J. Biol. Chem..

[27] Wark P.A., Lau R., Norat T., Kampman E. (2012). Magnesium intake and colorectal tumor risk: a case-control study and meta-analysis. Am. J. Clin. Nutr..

[28] Li H., Feng X., Li H., Ma S., Song W., Yang B. (2023). The supplement of magnesium element to inhibit colorectal tumor cells. Biol. Trace Elem. Res..

[29] Paravicini T.M., Chubanov V., Gudermann T. (2012). TRPM7: a unique channel involved in magnesium homeostasis. Int. J. Biochem. Cell Biol..

[30] Dai Q., Shrubsole M.J., Ness R.M., Schlundt D., Cai Q., Smalley W.E. (2007). The relation of magnesium and calcium intakes and a genetic polymorphism in the magnesium transporter to colorectal neoplasia risk. Am. J. Clin. Nutr..

[31] Lee B.Y., Ordovás JM, Parks E.J., Anderson C.A.M., Barabási A.L., Clinton S.K. (2022). Research gaps and opportunities in precision nutrition: an NIH workshop report. Am. J. Clin. Nutr..

[32] Sun S., Zhu X., Huang X., Murff H.J., Ness R.M., Seidner D.L. (2021). On the robustness of inference of association with the gut microbiota in stool, rectal swab and mucosal tissue samples. Sci. Rep..

[33] Jones R.B., Zhu X., Moan E., Murff H.J., Ness R.M., Seidner D.L. (2018). Inter-niche and inter-individual variation in gut microbial community assessment using stool, rectal swab, and mucosal samples. Sci. Rep..

[34] Segata N., Waldron L., Ballarini A., Narasimhan V., Jousson O., Huttenhower C. (2012). Metagenomic microbial community profiling using unique clade-specific marker genes. Nat. Methods..

[35] Franzosa E.A., McIver L.J., Rahnavard G., Thompson L.R., Schirmer M., Weingart G. (2018). Species-level functional profiling of metagenomes and metatranscriptomes. Nat. Methods..

[36] Fan L., Yu D., Zhu X., Huang X., Murff H.J., Azcarate-Peril M.A. (2021). Magnesium and imidazole propionate. Clin. Nutr. ESPEN.

[37] Fan L., Zhu X., Sun S., Yu C., Huang X., Ness R. (2022). Ca:Mg ratio, medium-chain fatty acids, and the gut microbiome. Clin. Nutr..

[38] Webb M. (1953). Effects of magnesium on cellular division in bacteria. Science.

[39] Veerkamp J.H. (1977). Effects of growth conditions on the ion composition of Bifidobacterium bifidum subsp. Pennsylvanicum. Antonie Van Leeuwenhoek.

[40] Hébert E.M., Raya R.R., de Giori G.S. (2004). Nutritional requirements of Lactobacillus delbrueckii subsp. lactis in a chemically defined medium. Curr. Microbiol..

[41] Ryazanova L.V., Rondon L.J., Zierler S., Hu Z., Galli J., Yamaguchi T.P. (2010). TRPM7 is essential for Mg2+ homeostasis in mammals. Nat. Commun..

[42] Hoenderop J.G.J., Bindels R.J.M. (2005). Epithelial Ca2+ and Mg2+ channels in health and disease. J. Am. Soc. Nephrol..

[43] Hermosura M.C., Nayakanti H., Dorovkov M.V., Calderon F.R., Ryazanov A.G., Haymer D.S. (2005). A TRPM7 variant shows altered sensitivity to magnesium that may contribute to the pathogenesis of two Guamanian neurodegenerative disorders. Proc. Natl. Acad. Sci..

[44] Yu S., Wang X., Li Z., Jin D., Yu M., Li J. (2024). Solobacterium moorei promotes the progression of adenomatous polyps by causing inflammation and disrupting the intestinal barrier. J. Transl. Med..

[45] Seelig M.S., Altura B.M., Altura B.T. (2004). Benefits and risks of sex hormone replacement in postmenopausal women. J. Am. Coll. Nutr..

[46] Avelar-Barragan J., DeDecker L., Lu Z.N., Coppedge B., Karnes W.E., Whiteson K.L. (2022). Distinct colon mucosa microbiomes associated with tubular adenomas and serrated polyps. Npj Biofilms Microbiomes.

