# Supplementary material for: Magnesium treatment increases gut microbiome synthesizing vitamin D and inhibiting colorectal cancer: results from a double-blind precision-based randomized placebo-controlled trial
Source: Am J Clin Nutr. 2025 Sep 9;122(5):1185–94. doi: 10.1016/j.ajcnut.2025.09.011 (PMC12799435; doi:10.1016/j.ajcnut.2025.09.011)
Supplement: multimedia component 1 [file mmc1.docx]

**Magnesium Treatment Increases Gut Microbiome Synthesizing Vitamin D and Inhibiting Colorectal Cancer: Results from a Double-Blind Precision-based Randomized Placebo-Controlled Trial**

First author: Elizabeth Sun

**Supplemental Table 1: Baseline Relative Abundance by Sex**

|  | **Female (n = 99)** | | | | **Male (n = 127)** | | | | |  |
| --- | --- | --- | --- | --- | --- | --- | --- | --- | --- | --- |
| **Bacteria** | **Mean** | **STD** | **Min** | **Max** | **Mean** | **STD** | | **Min** | **Max** | ***P* value** |
| Rectal mucosa |  |  |  |  |  |  |  | |  |  |
| *C. maltaromaticum* | 0.146 | 0.258 | 0.000 | 1.380 | 0.096 | 0.153 | 0.000 | | 0.602 | 0.07 |
| *F. prausnitzii* | 3.480 | 0.421 | 2.696 | 5.529 | 3.413 | 0.306 | 2.889 | | 4.704 | 0.17 |
|  |  |  |  |  |  |  |  | |  |  |
| Rectal swab |  |  |  |  |  |  |  | |  |  |
| *C. maltaromaticum* | 1.103 | 0.489 | 0.000 | 2.057 | 0.885 | 0.471 | 0.000 | | 2.045 | 0.001 |
| *F. prausnitzii* | 5.042 | 0.707 | 3.207 | 6.477 | 4.909 | 0.770 | 3.317 | | 6.241 | 0.17 |
|  |  |  |  |  |  |  |  | |  |  |
| Stool |  |  |  |  |  |  |  | |  |  |
| *C. maltaromaticum* | 1.195 | 0.408 | 0.000 | 2.164 | 1.195 | 0.449 | 0.000 | | 2.121 | 0.99 |
| *F. prausnitzii* | 5.296 | 0.566 | 3.784 | 6.527 | 5.409 | 0.534 | 3.240 | | 6.606 | 0.12 |

Abbreviations: *C. maltaromaticum*, *Carnobacterium* maltaromaticum; *F. prausnitzii,* Faecalibacterium prausnitzi; STD, standard deviation

| **Supplemental Table 2: Changes in *C. maltaromaticum* relative abundance by magnesium compared to placebo in rectal mucosa, swab, and stool, stratified by sex** | | | | | | | |
| --- | --- | --- | --- | --- | --- | --- | --- |
| **Participant Category**  **and Sample Type** | **Change in *C. maltaromaticum* Relative Abundance from Baseline** | | | | | | |
|  | **Mg Treatment** | |  | **Placebo** | | ***P*_1_** | ***P*_2_** |
|  | **(mean ± std)** | **%** |  | **(mean ± std)** | **%** |  |  |
| Females: (n=108) |  |  |  |  |  |  |  |
| Rectal mucosa | 0.006±0.325 | 4.85 |  | -0.011±0.325 | -6.54 | 0.79 | 0.74 |
| Rectal swab | 0.069±0.584 | 6.45 |  | -0.042±0.602 | -3.67 | 0.35 | 0.42 |
| Stool | 0.021±0.403 | 1.83 |  | -0.045±0.504 | -3.65 | 0.47 | 0.71 |
| Males (n=118) |  |  |  |  |  |  |  |
| Rectal mucosa | -0.004±0.208 | -5.33 |  | -0.009±0.265 | -7.73 | 0.92 | 0.39 |
| Rectal Swab | 0.148±0.602 | 17.17 |  | 0.065±0.522 | 7.20 | 0.45 | 0.60 |
| Stool | 0.074±0.395 | 6.14 |  | 0.062±0.406 | 5.26 | 0.87 | 0.55 |
| Females with *TRPM7* GG (n=64) |  |  |  |  |  |  |  |
| Rectal mucosa | 0.015±0.304 | 14.34 |  | -0.035±0.301 | -19.24 | 0.51 | 0.85 |
| Rectal swab | 0.239±0.624 | 24.06 |  | -0.177±0.588 | -14.70 | 0.01 | 0.02 |
| Stool | 0.152±0.406 | 13.52 |  | -0.109±0.513 | -9.27 | 0.04 | 0.04 |
| Males with *TRPM7 GG* (n=*7*9) |  |  |  |  |  |  |  |
| Rectal mucosa | 0.001±0.202 | 1.54 |  | -0.021±0.276 | -19.39 | 0.68 | 0.95 |
| Rectal swab | 0.200±0.617 | 22.25 |  | 0.036±0.578 | 4.06 | 0.26 | 0.13 |
| Stool | 0.071±0.424 | 6.10 |  | 0.071±0.355 | 5.93 | 1.00 | 1.00 |
| Females with *TRPM7* GA (n=44) |  |  |  |  |  |  |  |
| Rectal mucosa | -0.007±0.360 | -4.41 |  | 0.026±0.362 | 18.13 | 0.77 | 0.91 |
| Rectal swab | -0.141±0.461 | -12.15 |  | 0.151±0.581 | 14.36 | 0.07 | 0.12 |
| Stool | -0.147±0.337 | -12.30 |  | 0.051±0.487 | 3.93 | 0.13 | 0.06 |
| Males with *TRPM7* GA (n=39) |  |  |  |  |  |  |  |
| Rectal mucosa | -0.016±0.224 | -21.81 |  | 0.015±0.246 | 12.24 | 0.68 | 0.13 |
| Rectal swab | 0.034±0.568 | 4.36 |  | 0.113±0.422 | 12.09 | 0.63 | 0.19 |
| Stool | 0.082±0.322 | 6.22 |  | 0.048±0.486 | 4.13 | 0.80 | 0.21 |
| Generalized linear model were used: *P*1 not adjusted*; P*2 adjusted for age, sex, BMI and baseline level*.*  P for interaction between treatment and sex for C. maltaromaticum: 0.83 in mucosa; 0.82 in swab; and 0.93 in stool. *P* for interaction between treatment and *TRPM7* genotype among females: 0.97 in mucosa; 0.01 in rectal swab; and 0.003 in stool.  Abbreviations: *C. maltaromaticum*, *Carnobacterium maltaromaticum*; Mg, magnesium; *TRPM7, transient receptor potential cation channel, subfamily M, member 7*; STD, standard deviation | | | | | | | |

| **Supplemental Table 3: Changes in *F. prausnitzii* relative abundance by magnesium compared to placebo in rectal mucosa, swab, and stool, stratified by sex** | | | | | | | |
| --- | --- | --- | --- | --- | --- | --- | --- |
| **Participant Category**  **and Sample Type** | **Change in *F. prausnitzii* Relative Abundance from Baseline** | | | | | | |
|  | **Mg Treatment** | |  | **Placebo** | | ***P*_1_** | ***P*_2_** |
|  | **(mean ± std)** | **%** |  | **(mean ± std)** | **%** |  |  |
| Females: (n=108) |  |  |  |  |  |  |  |
| Rectal mucosa | -0.047±0.462 | -1.36 |  | 0.055±0.542 | 1.58 | 0.29 | 0.12 |
| Rectal swab | -0.027±0.715 | -0.55 |  | -0.182±0.770 | -3.58 | 0.27 | 0.38 |
| Stool | -0.139±0.478 | -2.63 |  | -0.084±0.494 | -1.59 | 0.56 | 0.54 |
| Males (n=118) |  |  |  |  |  |  |  |
| Rectal mucosa | 0.010±0.321 | 0.28 |  | 0.038±0.279 | 1.11 | 0.61 | 0.73 |
| Rectal Swab | 0.108±0.847 | 2.20 |  | 0.119±0.710 | 2.44 | 0.94 | 0.88 |
| Stool | -0.020±0.459 | -0.37 |  | -0.010±0.618 | -0.18 | 0.92 | 0.46 |
| Females with *TRPM7* GG (n=64) |  |  |  |  |  |  |  |
| Rectal mucosa | -0.007±0.348 | -0.21 |  | -0.060±0.454 | -1.72 | 0.60 | 1.00 |
| Rectal swab | 0.024±0.840 | 0.49 |  | -0.262±0.884 | -7.06 | 0.18 | 0.08 |
| Stool | -0.132±0.556 | -2.50 |  | -0.116±0.530 | -2.23 | 0.91 | 0.96 |
| Males with *TRPM7* GG (n=79) |  |  |  |  |  |  |  |
| Rectal mucosa | 0.016±0.291 | 0.47 |  | 0.029±0.278 | 0.87 | 0.84 | 0.58 |
| Rectal swab | 0.169±0.804 | 3.38 |  | 0.056±0.812 | 1.13 | 0.53 | 0.26 |
| Stool | -0.051±0.497 | -0.93 |  | 0.010±0.597 | 0.18 | 0.62 | 0.58 |
| Females with *TRPM7* GA (n=44) |  |  |  |  |  |  |  |
| Rectal mucosa | -0.104±0.592 | -2.91 |  | 0.228±0.623 | 6.54 | 0.08 | 0.06 |
| Rectal swab | -0.099±0.501 | -1.94 |  | -0.055±0.535 | -1.04 | 0.78 | 0.41 |
| Stool | -0.148±0.376 | -2.79 |  | -0.037±0.443 | -0.69 | 0.37 | 0.23 |
| Males with *TRPM7* GA (n=39) |  |  |  |  |  |  |  |
| Rectal mucosa | -0.004±0.385 | -0.13 |  | 0.054±0.287 | 1.56 | 0.59 | 0.23 |
| Rectal swab | -0.015±0.939 | -0.31 |  | 0.237±0.462 | 4.89 | 0.28 | 0.21 |
| Stool | 0.054±0.353 | 0.99 |  | -0.043±0.666 | -0.80 | 0.57 | 0.46 |
| Generalized linear model were used: *P*1 not adjusted*; P*2 adjusted for age, sex, BMI and baseline level*.*  P for interaction between treatment and *sex* for *F. prausnitzii*: 0.24 in mucosa; 0.61 in swab; and 0.38 in stool. *P* for interaction between treatment and *TRPM7* genotype among females: 0.12 in mucosa; 0.09 in rectal swab; and 0.33 in stool. | | | | | | | |

Abbreviations: *F. prausnitzii,* Faecalibacterium prausnitzii; Mg, magnesium; *TRPM7, transient receptor potential cation channel, subfamily M, member 7*; STD, standard deviation
